# Cardio-oncology research prioritisation in the United Kingdom: national surveys of health care professionals, patients and carers

**DOI:** 10.1186/s40959-026-00503-0

**Published:** 2026-05-20

**Authors:** Gerard M Walls, Allyson Arnold, David Austin, Alison R Fielding, Simon P Fisher, Christopher A Miller, Charlotte Manisty, Anna Olsson-Brown, Mark C Petrie, John A Snowden, Nick Hartshorne-Evans, Keith Wilson, Vasilena Zhecheva, Ninian N Lang

**Affiliations:** 1 The Johnston Cancer Research Centre, Queen’s University Belfast, Jubilee Road, Belfast, Northern Ireland UK; 2 Cancer Centre Belfast City Hospital, Belfast Health & Social Care Trust, Northern Ireland, UK; 3 British Cardiovascular Society, London, England, UK; 4 Academic Cardiovascular Unit, James Cook University Hospital, South Tees Hospitals NHS Foundation Trust, Middlesbrough, UK; 5 Population Health Sciences Institute, Newcastle University, Newcastle upon Tyne, UK; 6 Independent patient advocate, Surrey, England, UK; 7 NIHR-BHF Cardiovascular Partnership, University of Oxford, Oxford, England, UK; 8 Manchester University NHS Foundation Trust, Manchester, England, UK; 9 Division of Cardiovascular Sciences, University of Manchester, England, UK; 10 Barts Heart Centre, Barts Health NHS Trust, London, England, UK; 11 Institute of Cardiovascular Science, University College London, London, England, UK; 12 The Clatterbridge Cancer Centre NHS Foundation Trust, Liverpool, England, UK; 13 University Hospital Sussex NHS Foundation Trust, Brighton, England, UK; 14 British Heart Foundation Glasgow Cardiovascular Research Centre, School of Cardiovascular and Metabolic Health, University of Glasgow, Glasgow, Scotland, UK; 15 Division of Clinical Medicine, School of Medicine and Population Health, The University of Sheffield, Sheffield, UK; 16 Department of Haematology, Sheffield Teaching Hospitals NHS Foundation Trust, Sheffield, England, UK; 17 Pumping Marvellous Foundation, Preston, England, UK; 18 Liverpool Heart and Chest Hospital, Liverpool Heart and Chest Hospital, Cardiology, Liverpool, England, UK

**Keywords:** Cardiotoxicity, Cardio-oncology, Research strategy, National survey, Patient perception

## Abstract

**Background:**

The relevance of cardiovascular disease in people with a history of cancer has increased in parallel with dramatic improvements in cancer-specific outcomes. There is an urgent need to improve the evidence underpinning cardio-oncology practice. Research prioritisation is essential and needs to account for multidisciplinary healthcare professional (HCP) perspectives, as well as patients and carers.

**Methods:**

The NIHR-BHF Cardiovascular Partnership Theme in Cardio-Oncology conducted two UK-wide online surveys to identify research priorities. The first was distributed to HCPs with cardiology, oncology and haemato-oncology backgrounds. The second was distributed to patients and carers (PCs) with experience of cancer and/or cardiovascular disease. Surveys were co-designed by clinicians and patients.

**Results:**

HCP Survey: 127 responded; 53% prioritised research ‘during cancer treatment’. Immune checkpoint inhibitors and targeted therapies were identified as the highest-priority drug classes. Cardiac dysfunction/heart failure (53%) and myocarditis (22%) were priority cardiovascular toxicities of interest. The development of a cardio-oncology registry was marginally favoured over randomised trials. Prospective randomised open-label blinded endpoints (PROBE) designs were considered of similar priority to double-blinded placebo controlled trials. PC Survey: 267 responded. 54% were concerned about the impact of cancer treatment upon cardiovascular health. PC research priorities were: prevention of cardiac side effects (58%), long-term cardiac monitoring, and early detection of side effects. Willingness to participate in research was high.

**Conclusion:**

HCPs and PCs from the UK prioritised prevention and detection of cardiac dysfunction during and immediately following cancer therapy, particularly with agents such as immune checkpoint inhibitors and targeted therapies. These findings provide important strategy-setting insights for large-scale collaborative cardio-oncology studies.

**Supplementary Information:**

The online version contains supplementary material available at 10.1186/s40959-026-00503-0.

## Introduction

Dramatic improvements in cancer survival are, in large part, a consequence of the rapid evolution and introduction of novel cancer therapies [1]. Particularly in the context of these improved cancer-specific outcomes, much greater attention is now paid to the competing risks of cardiovascular morbidity and mortality in patients with active cancer and in cancer survivors [2]. Patients with a history of cancer are at increased risk from a range of cardiovascular conditions because of shared risk factors, such as smoking, obesity and inflammation [3]. The adverse cardiovascular effects of conventional chemotherapeutics, such as anthracyclines, have been widely acknowledged for many years [4]. The potential for adverse cardiovascular effects of newer cancer therapies is increasingly recognised but not always informed by robust evidence [5]. This means that strategies to predict, monitor, prevent and treat potential adverse cardiovascular effects of cancer therapies require further co-ordinated research [6].

In the context of this large unmet need in cardio-oncology research [7–9], it is particularly important to define research priorities and to consider the unique perspectives of medical practitioners with multi-disciplinary backgrounds in oncology, haemato-oncology and cardiology. It is critical that such research aligns with the priorities of patients and their carers and is conducted in a manner that maximises their participation [10]. Experts in the field have identified evidence gaps in cardio-oncology [7–9], and prior work has examined patients’ understanding of the potential for cardiovascular issues as a consequence of cancer therapy [11, 12]. However, research prioritisation-setting has been relatively lacking.

In order to formulate a national strategy for cardio-oncology research in the UK, the National Institute for Health and Care Research–British Heart Foundation (NIHR-BHF) Cardiovascular Partnership Theme in Cardio-Oncology conducted two cross-sectional assessments ofstakeholder perceptions and opinions regarding strategic research priorities.

## Methods

Two complementary UK-based surveys were developed (Supplementary Appendices 1–2). The first survey was designed for completion by healthcare professionals (HCPs) working in cardiology, oncology and haemato-oncology settings. The second was designed for completion by patients with a history of cancer and/or heart disease as well as carers of affected individuals (PCs). Online questionnaires were co-designed by clinicians (oncologists, haemato-oncologists and cardiologists) and patient representatives. In addition to circulation via the authors’ multidisciplinary networks, multi-professional cardiology, haematology and oncology networks agreed to disseminate the survey by email, newsletters and social media pages (September to November 2024; Supplementary Appendix 3).

## Results

### Healthcare professionals survey

There were 127 respondents to the HCP survey, of whom 44% had a background in oncology, 14% haemato-oncology and 34% cardiology. The majority of respondents were medical practitioners (75%) while 13% were nurses, 7% pharmacists and 6% were allied health professionals including echocardiographers, therapeutic radiographers and physiotherapists. All four UK nations (England, Scotland, Wales, Northern Ireland) were represented in the responses, with respondents from 58 different hospitals.

**Fig. 1:**
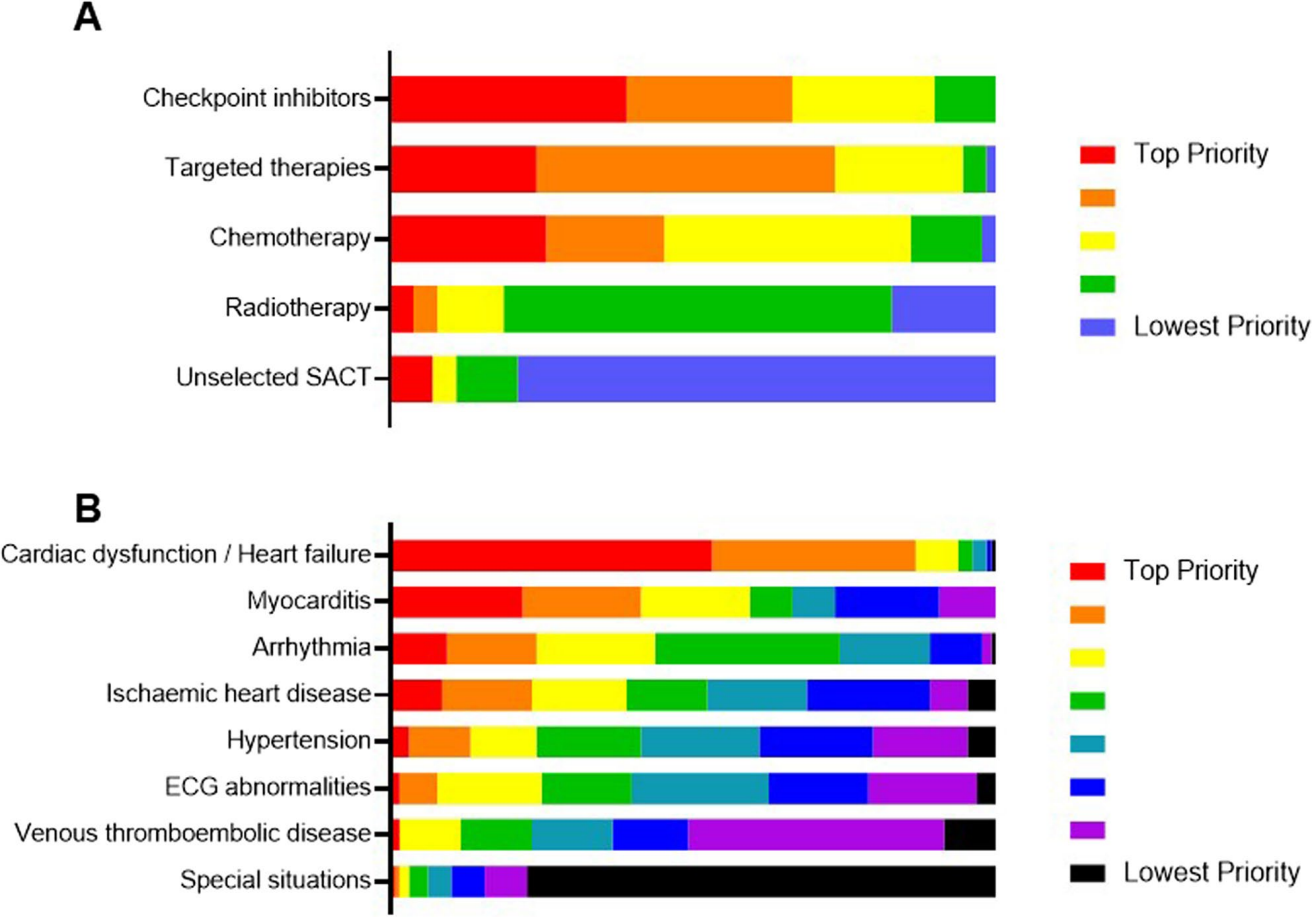
Research priorities reported by clinicians (*n* = 127), in relation to specific anti-cancer treatments (**A**) and subtypes of cardiac adverse events (**B**), ranked highest to lowest

When asked to consider the ‘phase’ of the cancer treatment journey upon which research should be prioritised, the majority of respondents selected ‘during treatment’ (53%), followed by ‘before treatment’ (37%) and ‘after treatment’ (10%). Cancer therapy treatment classes that were considered to be top priorities for cardio-oncology research were immune checkpoint inhibitors (39% top priority, 28% second priority choice) and targeted therapies (24% first priority, 49% second priority choice) while conventional chemotherapy (such as anthracyclines and platinum) was a lower priority (25% first priority, 20% second priority choice) (Fig. 1A). Of the potential cardiovascular adverse effects of anti-cancer therapy, ‘cardiac dysfunction/heart failure’ was by far the most common priority (53% first choice priority), followed myocarditis (22%) (Fig. 1B).

The development of a cardio-oncology registry was a top priority for 59% of respondents while cardio-oncology trials were a top priority for 42%. In terms of trial design, randomised, double-blinded controlled trials were considered to be of similar priority to prospective randomised, open-label with blinded endpoints (PROBE) trials. The top five most popular outcome measures were hospitalisation for CV reasons, followed by heart failure admission/urgent care, cardiovascular mortality, cancer therapy modification, and all-cause hospitalisation.

### Patient and carer survey

There were 267 respondents to the PC survey. The majority of respondents were aged 55 years or more (73% female; 92% White), and 87% either had a history of cancer (48%), were a family member or carer for a person with a history of cancer (32%), or both (7%). Most were either living with cardiovascular disease (73%), were a family member or carer for a person with cardiovascular disease (10%), or both (10%). Just under half (44%) had experienced a cardiovascular condition since completion of cancer treatment. Respondents considered that these cardiovascular conditions and co-morbidities were impacting their life ‘a lot’ (35%), ‘a little’ (50%) or ‘not at all’ (15%). Half of respondents (54%) were either ‘very concerned’ or ‘somewhat concerned’ about the impact of cancer treatment upon cardiovascular health while the minority (18%) were either ‘somewhat unconcerned’ or ‘very unconcerned’.

**Fig. 2:**
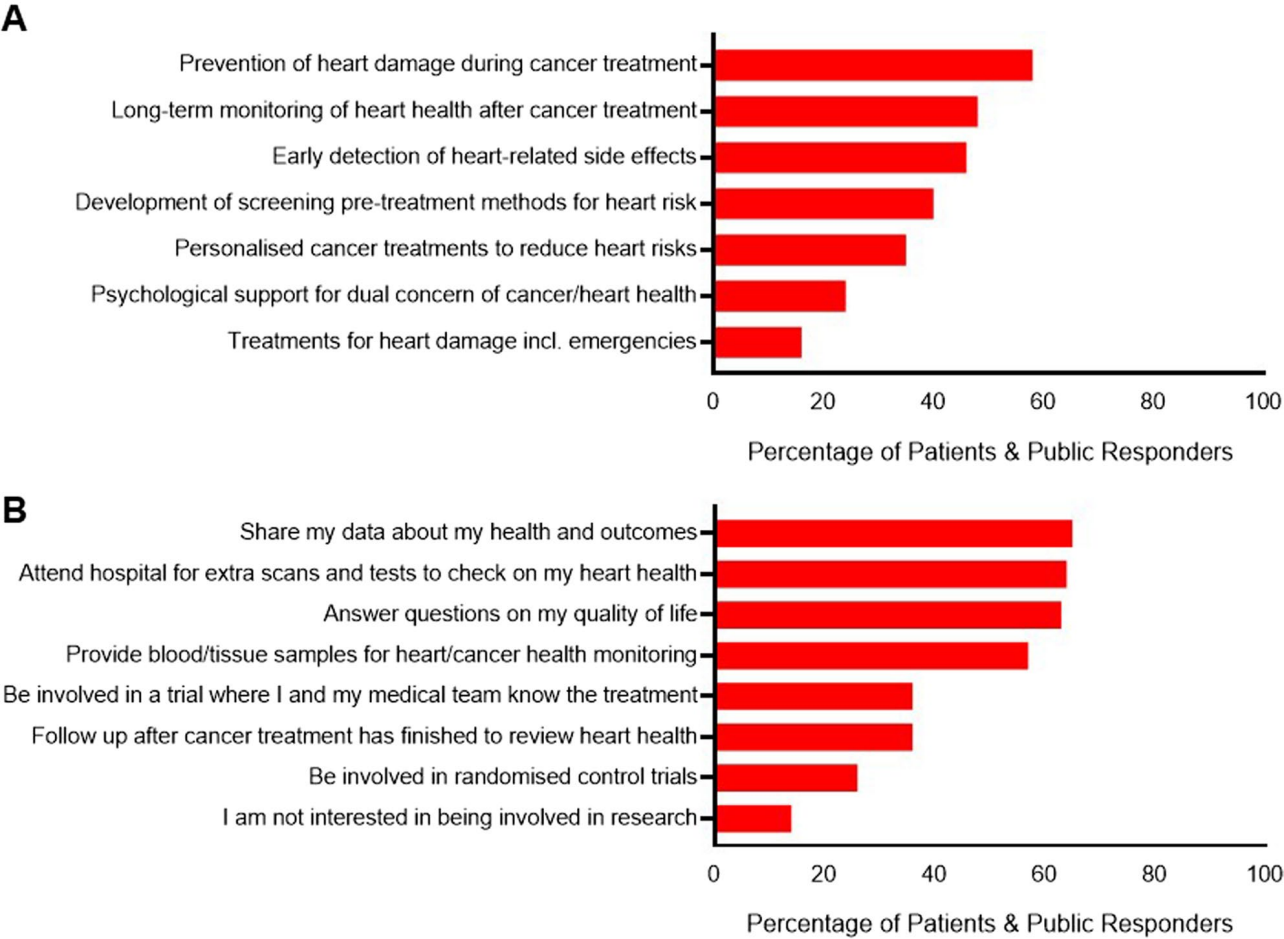
Prioritisation of cardio-oncology research topics as reported by patients and their carers (*n* = 267) when permitted to select a maximum of 3 options (**A**) and the types of common research activities they would readily participate in (**B**), ranked highest to lowest

Patients and carers prioritised ‘prevention’ of cardiac side effects of anti-cancer therapy most frequently (58%), followed by ‘long term monitoring of heart health’, ‘early detection of heart-related side effects’ and ‘development of screening methods for heart risk before cancer treatment’ (Fig. 2A). When asked to select their top priorities for research outcomes of interest ‘quality of life in the long-term’ and ‘avoiding short-term death from cancer or heart disease’ were most frequently selected. In terms of willingness to participate in cardio-oncology research, the majority of respondents would be happy to provide information relating to quality of life (63%), to attend additional hospital visits for study activities (64%), to share routine National Health Service data for research (65%) and to provide biological samples (57%) (Fig. 2B).

## Discussion

These two large, complementary surveys provide valuable insights to allow prioritisation in cardio-oncology research in the UK and internationally. It is of note that 90% of HCPs in the UK considered that current cardio-oncology research should focus on either the ‘before’ or ‘during’ cancer treatment phase rather than the post-treatment phase. However, by focusing efforts on these two earlier phases it would be anticipated that this will have positive downstream effects that ultimately translate into longer-term improvements in the cardiovascular health of cancer survivors. Consistent with the rapid evolution of cancer therapies [13, 14], research relating to immune checkpoint inhibitors and targeted therapies was a priority of surveyed HCPs. While research into conventional chemotherapies, such as anthracyclines, was identified as a lower priority by HCPs, this does remain an area of ongoing unmet need [15, 16]. It is likely that research focusing on these treatments will benefit from greater focus upon patients who are at higher risk for cardiac adverse effects in contrast to recent neutral trials conducted in lower-risk patients [17]. The cardiotoxic effects of anti-cancer therapy are diverse and can include ischaemic events, arrhythmia and hypertension [18–20]. However, the surveyed UK HCP community seems to be most focused upon research into treatment-associated cardiac dysfunction and heart failure. Consistent with the phenomenal growth and success with the use of immune checkpoint inhibitors [19] the need for further research into associated myocarditis was recognized by HCPs.

Although the development of a cardio-oncology registry was marginally prioritised over clinical trials by HCPs, the difference in enthusiasm for these was not large and may have reflected the perception of feasibility as much as it reflected genuine need. The UK Cardio-Oncology HCP community’s desire for randomised trials in cardio-oncology remains clear. Although double-blinded placebo-controlled trials are the bedrock of the evidence-base in ‘conventional’ cardiology, the HCP survey revealed strong support for PROBE designs in the cardio-oncology setting. In patients with cancer who may be undergoing complex therapeutic regimens, opportunities to conduct robust PROBE trials should not be discounted. Indeed, it is possible that trial recruitment and retention may be enhanced if the patient and clinician are aware of treatment assignment [21].

In the large UK-focused PC survey, over half of respondents expressed concern about the potential for cancer treatments to have adverse effects upon the heart. The prioritisation of research into ‘cardiotoxic effects’ and ‘early detection’ aligns with the priorities expressed by HCPs and, given this overlap, should be a major catalyst for research in these areas. Avoiding short-term death from cancer or heart disease is an all-encompassing priority but allowing patients to avoid serious side effects such as heart failure, to maintain independence and to improve their long-term quality of life will be key aims for future research. The capture of patient reported outcome measures should not be limited to the short-term. Indeed, Oncology studies have illustrated the value of long-term capture of these [22]. Gratifyingly, patients and carers showed willingness for participation in future research in keeping with opinions expressed in relation to other topics in cardiology research [23]. It remains vital that, having set initial priorities, they remain deeply integrated in the design of future cardio-oncology research studies and trials.

The geographical context of this cross-sectional exercise is important to consider. In contrast with most countries, in the UK non-surgical oncology is divided into Clinical Oncology, Medical Oncology and Haemato-Oncology. Physicians from the latter are responsible for the care of patients with blood and bone marrow cancers, whereas Clinical and Medical Oncology are concerned with solid tumours. In the UK, Clinical Oncologists treat with radiotherapy and systemic therapy, whereas Medical Oncologists utilise systemic therapies only. Therefore, these different sub-specialties differ slightly from the more commonly used ‘Medical Oncology’ and ‘Radiation Oncology’ titles. While these geographic differences do need to be taken into consideration, we believe that the core themes expressed by respondents remain relevant for cardio-oncology research prioritisation internationally. Furthermore, local replication of this can be considered where appropriate and feasible.

Secondly, international comparisons must account for differences in cancer incidence trends. Indeed, priorities may differ in regions where specific cancer types have relatively greater incidence, such as in East Asia where there is a higher relative incidence of gastric cancer and Australia, where skin cancer remains a dominant concern [24]. In the UK, healthcare is predominantly state-funded and, therefore, this funding and infrastructure model is likely to influence both prioritisation and perception of the feasibility of conducting specific types of research.

To our knowledge these complementary surveys represent the first attempts to capture the opinion of unselected Cardio-Oncology HCPs, and affected patients and carers. The surveys are strengthened by being co-designed by patients and clinicians.

While there was broad geographical representation from all four UK nations, there was limited representation from non-White patients, and men were under-represented. This limited some diversity of evaluable opinion, and suggests different methodologies are required to sample the broader population [25]. Whether these findings would be replicated in jurisdictions with alternate healthcare models (e.g. insurance-based or fee-for-service), and different cancer treatment patterns also requires investigation. As with most surveys of this type, there is an unavoidable and unquantifiable risk of participation, recall and survivorship bias in the PC responses. Finally, gaps in clinical outcomes or research outputs were not assessed as part of this study, rather, the results represent opinions of healthcare professionals and patient/public participants.

We believe that these surveys will provide meaningful direction to focus the prioritisation of cardio-oncology research. While the surveys were conducted in the United Kingdom, many of the insights are of relevance to the broader international community. In addition to guiding cardio-oncology researchers, our findings should also be of value to funding bodies and institutions. These insights will assist in the triage of research support for topics that are valued simultaneously by patients, their carers and the clinical community.

## Conclusion

This dual survey provides the first UK-wide assessment of stakeholder perceptions and strategic research priorities in cardio-oncology from both clinician and patient perspectives. There was clear alignment on the need for research focused on preventing and detecting cardiac dysfunction during and immediately following cancer therapy, particularly with modern agents such as immune checkpoint inhibitors and targeted therapies. The findings provide important strategy-setting insights for further growth in focused, collaborative, cardio-oncology studies and trials that enable patients to benefit from impressive advances in anti-cancer treatment while minimising adverse cardiovascular effects.

## Supplementary Information

Supplementary Material 1.

## Data Availability

Sharing of the data will be considered upon request.
