# Supplementary material for: Cardio-oncology research prioritisation in the United Kingdom: national surveys of health care professionals, patients and carers
Source: Cardiooncology. 2026 May 20;12:90. doi: 10.1186/s40959-026-00503-0 (PMC13359712; doi:10.1186/s40959-026-00503-0)
Supplement: Supplementary file 1 — Supplementary Material 1. [file 40959_2026_503_MOESM1_ESM.docx]

**Supplementary Appendices**

**Appendix 1**

Survey on Clinical Research Priorities for UK Cardio-Oncology

*Demographic Details*

Please provide the following:

1. **Base Hospital (and University affiliation if applicable):**
2. **Clinical background** (select one):
3. Cardiologist (heart failure)
4. Cardiologist (cardio-oncology)
5. Cardiologist (other – please provide details in free text)
6. Haemato-oncologist
7. Oncologist (Clinical Oncology)
8. Oncologist (Medical Oncology)
9. Pharmacist (Oncology)
10. Pharmacist (Cardiology)
11. Nurse (Cardiology)
12. Nurse (Oncology)
13. Other
14. **Years of experience since graduation:**
15. 0–5 years
16. 6–10 years
17. 11–20 years
18. 21–30 years
19. 31–40 years
20. >40 years

*Research Priorities and Study Design*

1. **Rank the following groups in order of priority for DELIVERABLE UK cardio-oncology research:**
2. Patients BEFORE cancer treatment (e.g. baseline risk stratification, cardiovascular ‘optimisation’ etc)
3. Patients DURING cancer treatment (e.g. prevention of cardiovascular sequelae of cancer treatment, monitoring strategies, cardioprotection, ‘permissive’ cardiotoxicity etc)
4. Patients AFTER cancer treatment (e.g. long-term surveillance and risk stratification, cardiac rehabilitation etc)
5. **Please include below any additional comments on Question 4** (optional)
6. **Rank the following study/trial formats in order of preference for DELIVERABLE UK cardio-oncology research:**
7. Registry – electronic health record linkage follow-up only
8. Registry – prospective, in-person follow-up, plus electronic health record linkage
9. Interventional – prospective, randomised, open-label, blinded endpoint (PROBE) trial
10. Interventional – randomised double-blinded, placebo controlled trial
11. **Please include below any additional comments on Question 6** (optional)
12. **Rank the following cancer treatment classes you would prioritise for cardio-oncology research:**
13. Targeted therapies (e.g. VEGF TKIs, BRAF/MEK inhibitors, anti-HER2 therapies)
14. Immune checkpoint inhibitors (e.g. CTLA-4, PD-1 inhibitors)
15. Chemotherapy (eg anthracyclines, platinum agents)
16. Radiotherapy
17. Unselected (i.e. study enrolment not on the basis of specific cancer treatment)
18. **Please include below any other cancer treatment classes you would like to prioritise for cardio-oncology research, which are not listed in question 8**
19. **Rank the potential cardiovascular adverse effects of cancer treatment that you would prioritise for research:**
20. Arrhythmia
21. Cardiac dysfunction and heart failure
22. ECG abnormalities (e.g. QT interval prolongation)
23. Hypertension
24. Ischaemic heart disease
25. Myocarditis
26. Venous thromboembolic disease (DVT/PTE)
27. Special situations (e.g. pregnancy, pacemakers) - please specify in question below
28. **Please detail any special situations from question 10 below** (optional)
29. **Rank the following outcome measures you would prioritise for DELIVERABLE UK cardio-oncology research:**
30. All-cause hospitalisation
31. Hospitalisation for cardiovascular reasons
32. Heart failure hospitalisation/urgent care
33. All-cause mortality
34. Cardiovascular mortality
35. Quality of life
36. Cardiac imaging
37. Cardiovascular blood biomarkers
38. Cancer therapy modification/interruption because of cardiovascular reasons
39. **Please include below any other outcome measures you would prioritise for DELIVERABLE UK cardio-oncology research** (optional)

*Delivery*

1. **Have you led/contributed to any prospective clinical research study in the last 5 years at your site? - please click all that apply and provide further detail in question 15**
2. Registry
3. Clinical Trial
4. Other
5. **If you have led/contributed to any prospective clinical research study in the last 5 years at your site, please specify details below** (optional)
6. **Have you led/contributed to a prospective cardio-oncology research study in the past 5 years?** (if yes, please specify below)
7. **Do you have a partner from the ‘opposite’ specialty who would participate as a co-investigator if a study opened at your site?** (i.e. do you have a link cardiologist if you are a (haemato-)oncologist, or a (haemato-)oncologist, if you are a cardiologist). If yes, please specify below:
8. **If you are a trainee of your specialty, would you be interested in enrolling as the Associate PI for a Cardio-Oncology study in your hospital?**
9. Yes
10. No
11. I don't know what this is
12. N/A
13. **Would you be interested in contributing to the design & delivery of a future prospective UK Cardio-Oncology clinical study?**
14. Yes
15. No
16. **If you wish, please provide your name, contact details and any other comments below.**

**Thank you for participating in this survey.**

**Appendix 2**

Cancer and the Heart - Public Priorities Survey

*Background information*

**1.Has anyone close to you, including yourself, ever been diagnosed with cancer?**

1. Yes
2. No
3. Prefer not to say

**2. (If selected Yes to Question1) Which of the following best describes you?**

1. A person who has received/recovered from a cancer diagnosis
2. The family member or caregiver of a person who has received/recovered from a diagnosed with cancer
3. All of the above

**3.Has anyone close to you, including yourself, ever been diagnosed with a heart condition?**

1. Yes
2. No
3. Prefer not to say

**4. (If selected Yes to Question3) Which of the following best describes you?**

1. A person living with a heart condition
2. The family member or caregiver of a person living with a heart condition
3. All of the above

**5.What types of treatment have you (or the person you are caring for) had? (Tick all that apply)**

1. Chemotherapy
2. Immunotherapy
3. Targeted therapies - Anti-HER2 treatments (eg herceptin/trastuzumab or related agents)
4. Targeted therapies - VEGF inhibitors (eg sunitinib, pazopanib etc)
5. Targeted therapies - BRAF/MEK inhibitors (eg dabrafenib, trametinib etc)
6. Targeted therapies - ALK inhibitors (eg crizotinib, lorlatinib etc)
7. Targeted therapies - TKIs (imatinib, dasatinib etc)
8. Radiotherapy
9. Surgery
10. Haematopoietic cell transplantation (Allo, Autologous, CAR-T)

*About you*

*Please tell us a little about yourself. 
We will use this information to make sure we are reaching a wide range of people and to****better understand the needs****of different groups.*

**6. What is your age group?**

1. Under 18
2. 18-24
3. 25-34
4. 35-44
5. 45-54
6. 55-64
7. 65-74
8. 75 or older
9. Prefer not to say

**7. Which gender do you identify most with?**

1. Male
2. Female
3. Non-binary
4. Prefer not to say
5. Other (type)

**8. What is your ethnic background?**

1. White (English/Welsh/Scottish/Northern Irish/British)
2. Irish
3. Gypsy or Traveller
4. Any other White background
5. Mixed or Multiple ethnic groups (e.g., White and Black Caribbean, White and Asian)
6. Asian or Asian British (e.g., Indian, Pakistani, Bangladeshi, Chinese)
7. Black or Black British (e.g., African, Caribbean)
8. Arab
9. Any other ethnic group
10. Prefer not to say

**9. Do you have any physical or mental health conditions or illnesses lasting or expected to last 12 months or more?**

1. Yes
2. No
3. Prefer not to say

**10. (If selected Yes to Question9) Does your condition or illness/do any of your conditions or illnesses reduce your ability to carry out day to day activities?**

1. Yes - a lot
2. Yes - a little
3. No
4. Prefer not to say

**11. Where do you currently live?**

1. England
2. Scotland
3. Wales
4. Northern Ireland
5. Outside of the UK
6. Prefer not to say

*Awareness and concerns*

**12. Did you know that certain cancer treatments can affect heart health?**

1. Yes, I'm aware
2. I've heard a little about this
3. No, I didn't know this

**13.Have you (or the person you are caring for) experienced any cardiovascular problems since starting cancer treatment?**

1. Yes
2. No
3. I don’t know

**14. How concerned are/were you about potential heart-related side effects of cancer treatment?**

1. Very concerned
2. Somewhat concerned
3. Neither concerned nor unconcerned
4. Somewhat unconcerned
5. Very unconcerned

**15.What would be most important to you as, or if you were to become, a cancer patient? (Select maximum 3 options)**

1. Avoiding short term death from cancer or heart disease
2. Being able to stay on cancer treatment
3. Avoiding serious side effects like heart failure or diabetes
4. Avoiding acute hospital admission
5. Remaining independent
6. My quality of life on cancer treatment
7. My quality of life longer term

*Research priorities*

**16.Which types of research on heart health during cancer treatment would you find most valuable? (Select maximum 3 options)**

1. Development of screening methods for heart risk before starting treatment
2. Early detection of heart-related side effects
3. Prevention of heart damage during cancer treatment
4. Long-term monitoring of heart health after cancer treatment
5. Personalised cancer treatments to reduce heart risks
6. Treatments for heart damage including emergency situations
7. Psychological support for coping with dual concerns of cancer and heart health

**17. Please write any specific research question you think should be prioritised for cardio-oncology research. Please note that sharing your answer is NOT a guarantee that this question will be selected among the top priorities at the end of this project.**

*Research participation*

*The following question aims to provide an understanding of the level of interest in research participation among the population. 
Please be advised that responding to the question below does****NOT****put any obligation on you to take part in research.*

**18. What type of research would you be prepared to take part in? Please select all that apply.**

1. Sharing my data about my health and outcomes e.g. NHS records.
2. Being involved in randomised control trials - where who gets the trial treatments are decided randomly. Participants not on the trial treatment do get the normal standard of care.
3. Answering questions on my quality of life.
4. Attending hospital for extra scans and tests to check on your heart health.
5. Follow up after cancer treatment has finished to review heart health.
6. Being involved in a trial where I and my medical team knew what treatment I was getting.
7. Providing samples such as blood and tissue samples to enable researchers to look for markers of heart or cancer health/progression. This may include genetic information.
8. I am not interested in being involved in research.

**19. What are your primary concerns regarding cancer treatment and heart health? Please write a brief response.**

**Appendix 3**

UK Societies and Organisations Approached Regarding Survey Dissemination

British Junior Cardiologists' Association

British Oncology Pharmacists’ Association

UK Oncology Nursing Group

British Cardiovascular Society

British Society of Blood and Bone Marrow Transplantation

Anthony Nolan

Myeloma UK

Cardiomyopathy UK

British Cardio-Oncology Society

British Society Haematology

Association of Cancer Physicians

Royal College Radiologists

British Heart Foundation Clinical Research Collaborative

West of Scotland Cardio-Nephro-Oncology Research Group

British Society of Cardiovascular Magnetic Resonance

ImmunoOncology Clinical Network
